# Reducing sedentary time in adults at risk of type 2 diabetes: process evaluation of the STAND (Sedentary Time ANd Diabetes) RCT

**DOI:** 10.1186/s12889-016-3941-9

**Published:** 2017-01-14

**Authors:** Stuart J. H. Biddle, Charlotte L. Edwardson, Trish Gorely, Emma G. Wilmot, Thomas Yates, Myra A. Nimmo, Kamlesh Khunti, Melanie J. Davies

**Affiliations:** 1 School of Sport, Exercise and Health Sciences, Loughborough University, Loughborough, UK; 2 Leicester Diabetes Centre, Leicester General Hospital, University Hospitals of Leicester NHS Trust, Leicester, UK; 3 School of Sport, University of Stirling, Stirling, UK; 4 The NIHR Leicester-Loughborough Diet, Lifestyle and Physical Activity Biomedical Research Unit, Leicester, UK; 5 Institute of Sport, Exercise & Active Living, Victoria University, Footscray Park, Melbourne, VIC 8001 Australia; 6 Derby Teaching Hospitals, Derby, UK; 7 College of Life and Environmental Sciences, University of Birmingham, Birmingham, UK

## Abstract

**Background:**

Reducing sedentary behaviour may have important health implications. This study evaluated the potential enablers and barriers for outcomes of a randomised controlled trial (RCT) designed to evaluate a pragmatic education based intervention designed to reduce sedentary (sitting) behaviour in young adults at high risk of type 2 diabetes.

**Methods:**

Data were collected from participants in the intervention group immediately after an educational workshop addressing sedentary time and diabetes risk (*n* = 71), through phone interviews 6 weeks (*n* = 45) after the workshop, and at the conclusion of the 12-month trial (*n* = 10). The two education session facilitators were also interviewed about the intervention.

**Results:**

The RCT showed no difference in sedentary time at 12 months between intervention and control arms. The lack of behaviour change appeared not to be attributed to the workshops, which were well led and very favourably received according to feedback. However, factors contributing to this lack of behaviour change include lack of perceived health risk from baseline measures feedback; the preference to adopt physically active behaviours rather than to sit less; certain barriers to sitting less; motivational drift after the 3-month follow-up measurements where participants had no contact for a further 9 months; and, for some, unreliability of the self-monitoring tool.

**Conclusions:**

The workshop was well led and well received by the attendees but future interventions need to consider more contact with participants, discuss any specific benefits around simply standing to reduce sitting time, address the barriers to sitting less, and provide a more user-friendly and reliable self-monitoring tool.

**Trial registration:**

Current controlled trials ISRCTN08434554, MRC project 91409. Registered retrospectively on 22 February 2011.

## Background

Sedentary behaviour is defined as sitting or lying down, with low energy expenditure, during waking hours [1]. Those with higher levels of sedentary time have been shown to be at elevated risk of various non-communicable diseases, including type 2 diabetes [2], and sometimes this has been independent of the levels of moderate-to-vigorous physical activity [3]. However, recent data have also shown sedentary behaviour effects to be attenuated by physical activity or BMI levels [4].

Interventions to reduce sedentary behaviour have, until recently, been dominated by a focus on television (TV) or ‘screen time’ for young people, and these interventions show significant but small changes [5–7]. In the past few years, interventions to reduce sitting time in adults have emerged. These have included designs that focus on environmental changes, such as introduction of sit-to-stand desks in the workplace [8, 9], psychological approaches to change [10], and use of prompts [11, 12]. Changes have been noted but generally are small.

We conducted a randomised controlled trial (RCT) to evaluate a pragmatic, relatively ‘light touch’, education based intervention designed to reduce sedentary time in younger adults at risk of type 2 diabetes. This was titled ‘project STAND’ – *S*edentary *T*ime *AN*d *D*iabetes. The study protocol [13] and a report on the 12-month outcomes have been published [14]. Essentially, the RCT comprised intervention and control arms, with the former attending a pragmatic single 3-h group-based educational workshop, designed to be feasible within a primary care setting and based on the DESMOND [15] and PREPARE [16] structured education protocols, supplemented by a 6-week motivational follow-up phone call to see how participants were progressing with their behaviour change efforts. In addition, intervention participants were given a self-monitoring and prompting tool – the ‘Gruve’ (MUVE, Inc., USA: www.muveinc.com). The Gruve is a waist worn accelerometer which monitors, and provides feedback to the wearer, on time spent sedentary and in light and moderate-to-vigorous physical activity and provides a prompt (vibration) after prolonged times of inactivity. Data can be downloaded and viewed on the Gruve website.

Various biological, physiological, and psycho-social measures were taken at baseline, 3 months and 12 months. The primary outcome was change in sedentary behaviour after 12 months as assessed by the triaxial Actigraph GT3X accelerometer (Actigraph, Pensacola, Florida, USA), and participants also wore the activPAL3^TM^ monitor (PAL Technologies, Glasgow, UK) on the thigh. This allowed for an additional measure of sitting time and for feedback from the activPAL during the educational workshop.

Using intention-to-treat analysis, the 12-month outcomes showed that there was no significant change in sedentary behaviour. The intervention group (*n* = 94) and control group (*n* = 93) reduced their daily sedentary time by 17.4 min per day and 13.8 min respectively. The adjusted difference between the changes in the two groups was not significant. Data for physical activity, assessed objectively and by self-report, showed no significant change either. Selected characteristics of the overall sample are shown in Table 1.

**Table 1:** Selected characteristics of total RCT sample at baseline

	*n*	Mean (SD) or %
Age (years)	187	32.8 (5.6)
Gender (% female)	187	68.5
Ethnicity (% black and minority ethnic group)	187	19.8
BMI (kg/m^2^)	187	34.6 (4.9)
Obese (%)	187	84.5
Waist (cm)	187	103.3 (13.9)
Waist-hip ratio	187	0.88 (0.10)
Body fat (%)	187	40.6 (7.1)

RCTs, while accepted as the gold standard design for interventions, often report outcomes without providing information on how or why behaviour changed or did not change. The translation of intervention studies into policy and practice, or the implementation of such findings, can be hampered by a lack of good process evaluation data [17]. Process evaluations embedded in RCTs help define enablers and barriers to behaviour change and assisting in implementing findings in ecologically valid settings. Including a process evaluation is considered “a good investment” in the updated MRC guidelines on complex interventions because they help “to explain discrepancies between expected and observed outcomes, to understand how context influences outcomes, and to provide insights to aid implementation” ([18], p.4).

Moreover, as Huis et al. ([19], p. 2) recently stated, “understanding RCT results is also complicated by the use of intention-to-treat analyses. To provide unbiased comparisons among the treatment groups, individuals or clusters are analysed according to the group (experimental or control) to which they were originally allocated, regardless of whether they actually received the improvement strategy. Therefore, it is necessary to combine the strength of an RCT with a well-designed process evaluation”.

To this end, alongside the RCT, we conducted a process evaluation of the STAND RCT which, as reported, failed to show a significant difference in sedentary time reduction at 12 months between intervention and control groups. Hence, the aim of the process evaluation was to gather data from participants and workshop educators concerning the delivery of the workshop and participant behaviour change strategies with the objective of better understanding the trial outcome findings.

## Methods

The study was approved by the Nottingham National Health Service Research Ethics Committee in May 2010 (reference 10/H0403/13) and outcome data have been published [14]. The trial was registered on 22nd February 2011, and the first participant was consented for the RCT on 9th March, 2011. All participants signed written informed consent.

### Participants

We drew on four sets of data to use in the process evaluation (see Table 2). First, we requested all workshop participants (*n* = 71) to provide feedback immediately after the educational workshops. Second, we conducted progress phone calls with 45 participants in the intervention arm 6 weeks after they attended the educational workshop. We attempted to contact all 71 participants that had attended the workshop but the remainder did not answer on at least three occasions and did not return phone messages. These data can be used for process evaluation purposes, although it should be recognised that the phone calls took place early in the 12 month intervention so really reflect an assessment of the initial phases of the trial. We have labelled these interviews ‘participants (6 weeks)’.

**Table 2:** Details of process evaluation data collected, time lines, and themes addressed

	Workshop educators/leaders	Workshop evaluations by participants	Participants (6 weeks)	Participants (end)
	*N* = 2. Data collected after conclusion of trial.	*N* = 71. Data collected on completion of workshop.	*N* = 45. Phone interviews 6 weeks after attendance of workshop.	*N* = 10. Phone interviews at conclusion of trail (12 months).
Themes:				
Awareness of risk	✓			✓
Strategies to reduce sitting time		✓	✓	✓
Barriers to reducing sitting time		✓	✓	✓
Use of self-monitoring device			✓	✓
Delivery of workshop	✓	✓		✓
Workshop feedback and suggested improvements	✓	✓	✓	✓

The third set of data was from participants at the conclusion of the 12 month trial. We have labelled these interviews ‘participants (end)’. Invitations for telephone interviews were sent to 28 participants from the STAND intervention arm of which 12 initially agreed to be interviewed. Two of the 12 failed to answer the phone on several occasions. Reasons for non-participation by the remaining 16 were: a). declined (*n* = 1); b). no reply (*n* = 13); c). emails returned as unavailable (*n* = 2). At least two attempts were made to contact invited interviewees. The final sample, therefore, comprised 10 participants (*n* = 7 female) who were interviewed by telephone in 2012 by a member of the research team. Male participants ranged from 32 to 36 years of age with body mass index (BMIs) in the obese range (33.3–37.3 kg/m^2^). Females ranged from 27 to 38 years of age with BMIs also in the obese range (29.9–41.7 kg/m^2^). Quotes are labelled as male/female (M/F) and age in years (e.g., F27). Where participants had the same sex and age, letters were used to distinguish them.

Finally, interviews took place with the two primary workshop educator/facilitators (both female) (WEs). Both consented to take part in single face-to-face interviews on completion of the study in March, 2013. One workshop educator (WE1), aged 30, is trained in physical activity and health research, has a PhD, and has teaching experience. The other (WE2), aged 47, is a trained nurse with 15 years’ experience in health and health promotion. Both were formally trained in DESMOND structured education philosophy and practices.

### Post-workshop evaluations

Participants attending the workshops completed an evaluation sheet immediately after the workshop finished, asking a range of questions, including the best parts of the workshop, key messages, delivery and ideas for improvement. In addition, they were asked about the easiest and most difficult times to sit less in their daily lives.

### Interview guides

The 6-week follow-up phone call responses were recorded on a 4-page checklist, which were then transferred to a spreadsheet containing either numerical data or open-ended written responses. Interview guides were produced for the interviews with participants (end) as well as for workshop leaders.

For participants (6 weeks), questions were asked about the following:

1. Have you tried to reduce your sitting time? If so, what changes have you made and what are your plans for the future?
2. Have you downloaded the Gruve software and have you used the Gruve? If so, what was your experience? Numerous questions were also asked about how often it was used, whether it was still being used, and whether it helped to reduce and break up their sitting time.

For participants (end), questions were asked about risk perception (both for diabetes and the risk factor of too much sitting), views on reducing sitting, including how successful they felt their behaviour change was, what strategies they used, and what barriers existed. In addition, questions addressed the habitual nature of sitting and how to break this habit, and what could have improved the intervention.

For the workshop educators, questions addressed their training and preparation to lead the workshops, the resources and tasks used in the workshops, perceptions of good and not so good aspects of the workshops, why they thought there may not be much behaviour change, including the use of the self-monitoring tool, and any other issues they wanted to raise.

## Analysis

The educational workshop evaluation sheets were analysed by listing the key responses and frequencies of responses. All interviews for participants (end) and workshop educators were transcribed and read thoroughly so as to become immersed in the data and understand the individuals’ perceptions. Inductive analysis was used to identify and organize themes arising from the raw data, although many of the themes were natural progressions from the themes established in the interview guides. Data from the participants (6 weeks) was a mix of quantitative data, including categorical and nominal data (e.g., did you download the Gruve software? Yes/No; how many days a week did you wear the Gruve *initially*? Everyday, >3d/week, <3d/week, never), and open-ended responses. The latter were read thoroughly. Inductive analysis was used to identify and organize themes arising from these raw data. These were mainly the questions concerning the strategies adopted to reduce sitting time, behaviour change plans for the future, experience using the Gruve, and whether, and why, they might recommend the STAND intervention programme to others.

## Results and discussion

The interview guides and checklist yielded responses by participants to issues concerning the delivery of the workshop, and ways to improve the intervention, the self-monitoring device provided, awareness of risk (including risk of too much sitting), strategies attempted to reduce sitting, and barriers to sitting less. These are shown in Table 2, including where data were extracted from.

### Intervention feedback

Participants (end) were asked what they thought about the intervention, including the workshop sessions, attendance at measurement clinics, and the behaviour change strategies discussed. There was a clear positive response, including feeling more motivated and perceiving they had made efforts to change their behaviour. However, as discussed later, whatever changes were made could have been too small, not detected, not sustained, or even exaggerated in the interviews.

The workshops were well received. All 44 of the participants (6 weeks) who responded to the question about recommending the workshop to friends answered positively. The key themes from these interviews centred on the workshops being highly informative, and useful, as well as dispelling myths about diabetes. For example, one said it was a “good education; did not realise the consequences of sitting for long periods before workshop”. Data from participants (end) were also positive about taking part in the study, with one stating that “I know for me … it definitely did make a difference to our health … I didn’t realise how massive a lifestyle thing it is” (F27). Another said “it was a real positive influence on me” (F38c), while one provided more tangible feedback about the effects of the study, alongside being happier in a new job: “I was actually spurred on by it, whatever your records say I have lost a stone since then”. Workshop attendees had very few ideas for improvement to the workshop content and delivery but more role modelling of less sitting during the workshop was suggested.

A clear theme concerned the motivational effect of the measurement clinics and the length of time between the 2^nd^ (3 month) and final (12 month) testing clinics. It was clear that some participants used the evaluation measurement clinics as goals to improve their behaviours and outcomes. But at the same time, several expressed the view that motivation faded after 3 months and that the 12 month clinic was too far off and with no prompting and contact taking place in between. Greater contact was certainly something that was seen to be potentially very helpful to boost any changes in behaviour.

The workshop evaluations by participants reported that the ‘best bits’ of the workshop were information on diabetes and the atmosphere of the workshop (see Table 3). The key messages of ‘sit less’, ‘move more’, ‘stand more’, and the risks of diabetes were mentioned in all workshop evaluations.

**Table 3:** Workshop feedback on ‘best bits’

Best bit	Frequency of responses
Information on diabetes	16
Workshop atmosphere	11
Receiving personal data on sitting levels and health	9
Interactive style and personalisation	5
Behaviour change concerning sitting and risks	4
The Gruve and self-monitoring	4

### Use of self-monitoring

The study employed a self-monitoring tool, the Gruve, as explained earlier. Self-monitoring and prompting are enablers of change and have been identified as successful behaviour change techniques in health contexts [20] and in sedentary behaviour interventions [21].

Interviews with participants (both end and 6 weeks) showed that they were generally quite positive about the device and certainly felt that it acted as an enabler and prompt to sit less. Summary data on the use of the Gruve from the interviews with participants (6 weeks) are shown in Table 4. From 31 participants (6 weeks) who provided comments, 77% had something positive to report about the Gruve. It was common to say that it acted as ‘a reminder’. Comments from participants (end) suggested that this led to greater automaticity whereby the use of the Gruve helped sitting less become more of a habit.

**Table 4:** Data concerning use of the Gruve self-monitoring device [data from participants (6 weeks) interviews]

Question	Categories	Responses (% of those providing responses)
How many days used initially?	Everyday	26 (90%)
	>3d/week	1 (0.4)
	<3d/week	1 (0.4)
	Never	0
How many days used now?	Everyday	13 (45)
	>3d/week	4 (14)
	<3d/week	1 (0.4)
	Never	10 (35)
Have you checked your data online?	Yes	22 (76)
	No	7 (24)
How many days do you log on?	>1/week	15 (65)
	<1/week	5 (22)
	Never	3 (13)
Is the vibration function useful?	Yes	25 (83)
	No	5 (17)
Did it help?	Yes	25 (83)
	No	5 (17)
Were you self-conscious wearing it?	Yes	5 (17)
	No	25 (83)

Responses from participants (6 weeks) showed that 64% attempted to download the Gruve software. But it was also clear that logistical problems prevented the Gruve from working effectively. From 31 participants (6 weeks) who provided comments, 42% stated that they had problems with the device or were negative about its use. From these interviews, and those conducted with participants (end), problems included computer synchronisation issues, incompatible computers, website navigation problems, device malfunction, battery life being too short, charging issues, finding it confusing, finding it uncomfortable, and lost devices. From the responses provided by participants (6 weeks), 90% said they used the Gruve everyday initially, but this fell to 45% at the time of the interview at 6 weeks (see Table 4).

Overall, however, it seems that the Gruve was perceived as a positive element of the study and should have helped participants in their quest to reduce their sitting time. Ways of optimising its use, including battery life, improving its reliability, and ease of accessing feedback, should be sought. Moreover, modern devices are tackling these issues, and this field is moving rapidly [22].

### Awareness of risk

The STAND intervention recruited people at risk of type 2 diabetes based on factors such as a high BMI and/or family history of T2DM or cardiovascular disease. Psychological frameworks underpinning the intervention, such as the Commonsense Model of Illness [23], suggest that a key component is risk appraisal. The interviews (end) probed three sources of risk information. The first concerned participants’ reaction to receiving a letter from their GP (general practitioner/family doctor) saying that they are at risk of type 2 diabetes, the second was how they reacted to their baseline test results which were presented to them at the workshop, and the third was whether they had considered that too much sitting was a health risk.

Results showed that most participants were not surprised by their GP’s letter. Most were aware of their risk of diabetes through family history or recognition of their weight status. Only one participant declared ‘shock’ at the letter, having expressed the view that they had not really thought about being at risk. The letter then created different reactions, ranging from not taking any action, feeling that their awareness had increased, to changing their dietary and physical activity patterns. However, most stated that they did not change their views of their health, probably because most knew of their elevated risk status anyway.

**Fig. 1:**
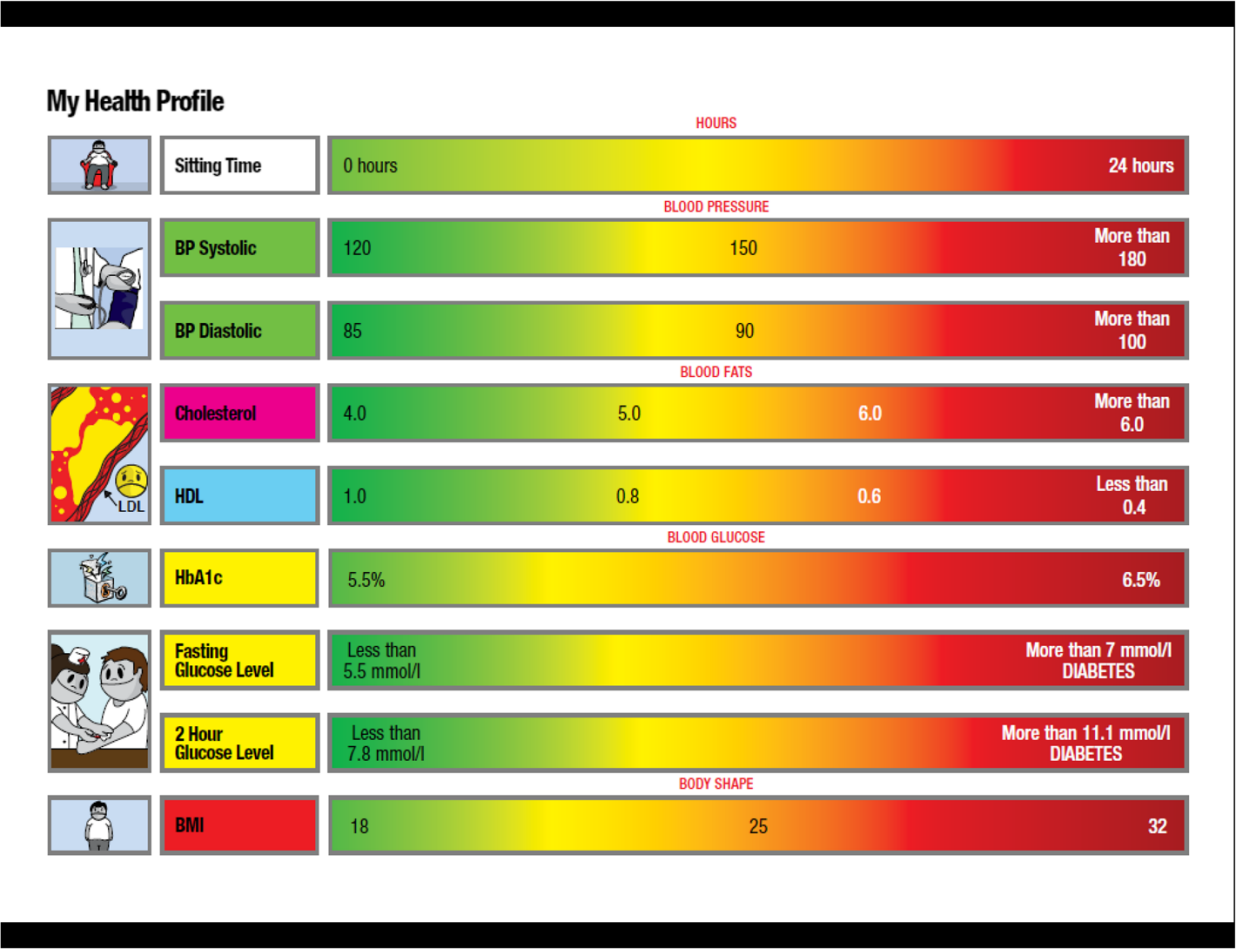
Health feedback chart used in workshops

A comprehensive health assessment, including blood tests, was conducted at the trial baseline clinic. These were sent to all participants (intervention and control) following the baseline clinics and they were used in the educational workshops as a point of learning and discussion. Attendees plotted their health results (i.e., BMI, glucose, cholesterol, blood pressure) on an attractive colour coded feedback chart, highlighting the risk zones of their health variables [risky (red), neutral (orange) and healthy (green) zones] (Fig. 1). In being asked how they reacted to their results, participants mainly expressed the view that they either couldn’t remember much about the results or they felt their awareness was increased. Only two people said that they had reacted strongly, one by expressing real concern about her cholesterol levels and another saying that her perspective had completely changed. Specifically, she said that previously she had seen diabetes as something she would have to deal with later in life, but now was motivated to take action sooner. To that end, she had lost weight and said the project had “made a huge difference to my perception” (F38c).

The workshop educators said it was clear that many participants saw their feedback profiles as essentially healthy. As one educator (WL1) expressed it “after looking at their health things and they saw their blood glucose was fine, their cholesterol was fine, and their blood pressure was fine, well ‘why am I at risk’? ‘I’m not at risk, clearly’.” Related to this was the view expressed that obesity is becoming the norm, and if there is no obvious sign of risk, why change?

Finally, participants were asked about the risk factor of too much sitting. Given the lack of success in bringing about changes to sitting time in the intervention at 12 months, it is important to understand how participants felt about this behaviour. There were two main themes that emerged. One is best described as ‘logical’ in so far as participants expressed the view that it was logical, maybe even obvious, that too much sitting will be bad for you. Several people recognised that their own lifestyle comprised sitting at work, TV viewing in the evening, and travelling in a car – all typical sedentary behaviours. Sitting at work was seen as particularly problematic with a perception that little could be done to change that. The second theme was that many referred to too much sitting and low levels of physical activity as interchangeable. To reduce sitting, many saw the alternative as getting up and moving around rather than continuing their behaviour standing. Indeed, one stated that she “didn’t really understand the standing thing” (F38b), while another felt that standing may not be enough to make much of a difference. “At the time (of the workshop) it seemed quite bizarre that standing up every so often could alter the fact or the outcome …” (F35). Others said that too much sitting had never been on their minds. Overall, little mention was made of the risk to developing type 2 diabetes, but rather to general health.

Overall, therefore, given that the trial itself did not bring about meaningful reductions in sedentary behaviour for the intervention participants compared to those in the control group, it must be concluded that either perceptions of risk were too variable, or simply did not translate into behaviour change. Psychological theory suggests that perceptions of risk should have some influence on behaviour, or at least intentions to change behaviour [24]. Participants in this trial may have changed their intentions but they did not change behaviour. Theoretical approaches relevant to this include the Health Belief Model (HBM) [25], Protection Motivation Theory [26], and the Commonsense Model of Illness [23]. For example, the HBM states that the likelihood of taking action (for one’s health) will partly be a function of the perception of susceptibility and seriousness of the health condition. This was not fully tested in our process evaluation and needs elaboration in future studies. Nevertheless, the theoretical approaches listed here are heavily focussed on cognitive processing, yet contemporary thinking suggests that affective reactions should also be considered for behaviour change. It was suggested many years ago that adopting physically active lifestyles might initially be driven by concerns about health, but behavioural maintenance will be more strongly associated with affective reactions to the changes being made (e.g., likes, dislikes, enjoyment) [27]. For these reasons, even if risk perception was evident in our participants, it may not have been translated into behaviour change if affective responses to sitting less were not particularly positive, see [28]. These notions require further testing.

### Views on reducing sitting

Participants (end) were asked whether they felt they had made any changes to their behaviour across the trial, and participants (6 weeks) were asked if they had tried to reduce their sitting during the initial phase of the trial and, if so, how?

While some participants mentioned changes to diet, most spoke either about trying to sit less or move more or, in some cases, both. The key strategies mentioned are shown in Table 5. There were 18 strategies mentioned to sit less with eight strategies to move more. It is clear from this table, and from the interviews, that people thought as much, if not more, about increasing their physical activity than reducing their sitting time, or breaking sitting time, by standing.

**Table 5:** Behaviour change strategies reported as attempted by participants

Strategies to ‘sit less’	Participants (6 weeks) *n* = 45	Participants (end) *n* = 10	Strategies to ‘move more’	Participants (6 weeks) *n* = 45	Participants (end) *n* = 10
Move printer to other side of the room	1	1	Walk	17	5
Stand more in work meetings	1	2	Park car further away and walk	1	1
Cleaning/housework	1	1	Gym	6	3
Stand on bus	1	1	Swimming		1
Replace sitting with various ‘activities’ (tasks)	1	1	Running	2	
Stand at work (e.g., to read)	1	1	Exercise	1	
Move waste bin away from desk		1	Exercise during TV adverts	1	
Use laptop on elevated surface	1	1	Active video games	1	
Stand while talking on phone	1	3			
Stand during TV breaks	6	1			
Moved TV out of bedroom	1				
Reduce or turn off TV	5				
Reduce time on laptop	1				
Stand more to eat	2				
Stand to play computer games	1				
‘Pottering’	1				
Manual washing up	1				
Get outside more	2				

The strategies for moving more were mainly walking (e.g., during lunch time at work or in the evening from home) or more structured exercise, such as attending a gym or going swimming. From the participant (6 weeks) data all but one said they had tried to reduce their sitting time and, as Table 5 shows, many of the commonly suggested strategies were attempted. Many involved television viewing, such as a general reduction, standing during breaks and removing it from a bedroom. Only one participant mentioned that attempting to reduce sitting was more difficult than they had envisaged, with several claiming it was easier to do that they had thought. When asked about their behaviour change plans for the future, 38 participants (6 weeks) provided comments, with only 11% of these referring to strategies to sit less. This is in contrast to 45% mentioning physical activity. Other comments were simply general statements about desired health outcomes.

From the participant (end) interviews, one person was clear in preferring to find activities or tasks to do rather than sit less through standing. She found this the “easiest way for me” (F27). One person was quite clear about this: “I’d rather be moving than standing” (F38a), while one was stronger in her views by stating that it was “a bit daft to stand” (F38b)! Others preferred to mix up the behaviours, such as walking to the shops more and standing in work meetings (M32). Some strategies to sit less met with failure. One person stated that they tried to use their laptop on an elevated surface, but soon found it awkward and unsuccessful (F38b).

The post-workshop evaluations showed that the perceived easiest times to reduce sitting were at home (*n* = 16), in the evening (*n* = 15), at work during breaks (*n* = 13), and during TV viewing (*n* = 9). These findings may reflect contexts that allow more choice.

The physical activity behaviours attempted were predictable. Walking was a popular choice as this may reflect the relative ease of fitting it in at lunchtimes at work or in the evenings. It can also be used for instrumental purposes, such as light shopping. While gym membership was also a strategy, some hinted at a lapse in involvement or declining motivation.

Overall, these findings suggest that strategies to sit less had been attempted and many of the traditional suggestions had been tried. However, there was a significant number who felt more inclined to increase their physical activity. While this, in itself, will be beneficial for health, it may lead to the situation where limited or very small changes in sitting time are evident. For example, a 20 min lunch time walk may be seen by an individual as a good attempt to improve their health, but then sit for the remainder of the day at work with little change in their sedentary time.

When putting these findings alongside the outcomes of the STAND trial, it seems that no measurable changes were noted in either sitting time or physical activity, so if the accounts presented here are reflective of the other participants, whatever changes were made were too small, not sustained, not detected by the accelerometers (e.g., swimming, cycling), or even exaggerated in the interviews, possibly due to a social desirability bias. Moreover, some of the statements concerning physical activity did appear somewhat ‘aspirational’.

### Barriers to reducing sedentary behaviour

From interviews with participants (end), there appeared to be three main themes emerging on barriers to sitting less. These concerned the participants’ work context, feelings of tiredness, and the inappropriateness of not sitting in some contexts.

It was often stated that to sit less at work was not easy, or close to impossible. This is despite discussing possible changes that could be made in the workshop. Interestingly, one participant was aware of standing desks as they knew a colleague had one because of back problems. However, the participant did express concerns that their employer was highly unlikely to purchase these as routine. Others simply said it was not really possible to stand much at work as they were employed for desk work. Moreover, the post-workshop evaluations showed that the perceived most difficult time to reduce sitting was at work (*n* = 17). Clearly, a better job at communicating with employees and employers, and demonstrating, how standing can be incorporated into jobs, including desk jobs, without loss of productivity is now required. When standing or moving alternatives were suggested, it was said that prompts are often required to remind people.

Related to work place sitting constraints was the theme concerning the perceived inappropriateness of standing in work meetings. It is often proposed that people could break up their sitting time by standing in business meetings. However, participants said it was “embarrassing to stand in meetings” (F27), that standing in meetings was “weird” (M32), another was uncomfortable standing in meetings (F38c), while one said “I can’t stand in a big room full of people” (F38a). Clearly there are perceived social constraints on this behaviour, but ones that may get broken down over time if more people adopt standing breaks in meetings. But for the purposes of this process evaluation, it was clearly a suggested strategy that did not work well.

The third barrier to sitting less was feelings of tiredness, usually at the end of the day. One also expressed the view that they wanted “me time” (F27) – a comment suggesting a preference for sedentary behaviour. This is supported by a view that laziness was a factor and, later in the evening, they simply “want to collapse and do nothing” (M32). This might reflect the importance of targeting certain times of the day for less sitting. Early to late evening may be low priority for this.

A further interesting issue that was raised, although only by one person, was whether small changes required more effort than larger ones. While this seems contradictory, the argument was that many small changes required more thought while one larger change allowed for better focus. For example, the participant (F27) said “… exercise in adverts (break), grab some beans and do some weights, or do some sit ups … you think that’s never really going to happen”. For them, it was more realistic to exercise at a gym, suggesting it was a more substantial thing to do. The small changes often suggested, such as exercising in TV advert breaks, seemed quite unrealistic for this one person. This is something that should be tested in future studies, perhaps through further interviews, or testing whether more ‘nudge’ based interventions might make both larger and smaller changes easier to do.

### Views expressed by workshop educators

From the structure of the interview guides, there are three main themes derived from the interviews with the two workshop educators: a). the training of the workshop educators, b). the workshop themselves, including resources used, and c). views about any noticeable change in sitting time from the intervention group.

The training of the workshop educators was seen as thorough and professional. Both were formally trained in DESMOND and had developed experience prior to the STAND workshops. They were confident in their roles.

The workshops were seen positively. It was felt that participants showed a high level of engagement and some participant “light bulb moments” were recalled by WE2. A key feature was working through the participant’s own health data from their baseline tests and activPAL sitting time data. This was seen as very important by the educators, and it lead to good discussions concerning possible sedentary behaviour reduction strategies. However, views about the use of the Gruve self-monitoring and prompting device were mixed. It was recognised that several problems existed, as identified by the participants. The workshop duration of three hours was seen to be about right.

It was suggested to the workshop educators that initial data analysis had shown little or no change in behaviour from the STAND RCT. Reasons offered for this included job restrictions and social norms that expect sitting. In addition, it was clear that many participants saw the feedback profiles of their biochemical tests as essentially healthy (see earlier). Moreover, it is suggested that obesity is becoming the norm, and so there is no obvious sign of risk and possibly no perception of the need to change if all other health variables are within the ‘normal’ range. Finally, the leaders of the workshops felt that more follow up and support for participants might have led to greater behaviour change.

## Conclusions

This process evaluation used four sets of data: workshop evaluation forms, intervention participant phone interviews after 6 weeks and 12 months, and interviews with two workshop educators. From this, five key conclusions are evident:

1. The workshops were led by well-trained educators. Participants liked the workshops and felt they were highly informative and useful. A lack of difference in behaviour change in the RCT between intervention and control arms appears not to be associated with the reaction of participants to the intervention workshops.
2. While the 3-month measurement clinic acted as an incentive to improve results, the 12-month clinic was seen as too far in the distance to assist motivation. However, the intention of the clinics was not, of course, to affect motivation. But more contact between clinics would have been helpful.
3. The Gruve self-monitoring tool was viewed favourably by participants but many also reported problems. It has good potential to assist prompting of behaviour change, but this device needs longer battery life, better reliability and easier ways to access feedback.
4. While perception of risk was somewhat evident, mainly through recognition of weight status and family history, feedback on personal health data at the workshops may have contributed to a perception from the participants that some had essentially healthy profiles. This may have contributed to lowered motivation for behaviour change.
5. Strategies to ‘reduce sitting’ often involved additional physical activity rather than targeting sitting per se. This may not have led to real reductions in sitting if the physical activity was moderate-to-vigorous and merely replaced previously ‘light physical activity’. In other words, activity increased with no commensurate change in sitting. We cannot be sure about this, but it is plausible. Equally possible is that whatever changes were made were not perceived by participants, not detected by the assessment devices, were not sustained, or were exaggerated in the interviews.
6. Barriers to sitting less included participants’ work context, feelings of tiredness at the end of day (when sitting is more likely), and perceived inappropriateness of not sitting in some contexts.

Based on this evidence, the failure of the STAND RCT to elicit significant behaviour change at 12 months between intervention and control arms [14] can probably be attributed to a certain lack of perceived risk from baseline measurement feedback; the preference to adopt physically active behaviours rather than to sit less; certain barriers to sitting less, including workplace constraints, fatigue at key points in the day, and social conventions; motivational drift after the 3-month testing clinic where participants had no contact for a further 9 months; and, for some, unreliability of the self-monitoring tool. The lack of behaviour change does not seem associated with the intervention workshops, which appeared to be well led and very favourably received, although recruitment was an issue with only 76% attending [14]. Replication and extension of this RCT needs to consider more contact with participants, more focus on addressing the message of ‘sit less’ in the context of the benefits of simply standing and the possible benefits to health. At the time of these workshops in 2010 we had less evidence than we have now and participants may have been less aware of the benefits. In addition, explicitly addressing the barriers to sitting less is required, as well as providing a more user-friendly and reliable self-monitoring tool.

## Abbreviations

BMI: Body mass index
CVD: Cardiovascular disease
DESMOND (programme): Diabetes education and self-management for ongoing and newly diagnosed
GP: General practitioner (family physician)
PREPARE (trial): Pre-diabetes risk education and physical activity recommendation and encouragement
RCT: Randomised controlled trial
STAND (project): Sedentary time and diabetes
T2DM: Type 2 diabetes mellitus
TV: Television
